# Micro-Feedback Training:Learning the art of effective feedback

**DOI:** 10.12669/pjms.336.13721

**Published:** 2017

**Authors:** Najma Baseer, Usman Mahboob, James Degnan

**Affiliations:** 1 Dr. Najma Baseer, MBBS, PhD. Assistant Professor in Anatomy, Institute of Basic Medical Sciences (IBMS), Khyber Medical University, Peshawar, Khyber Pakhtunkhwa, Pakistan; 2 Dr. Usman Mahboob MBBS, MPH, DHPE, FHEA. Assistant Professor in Medical Education Institute of Health Professions Education & Research Khyber Medical niversity, Peshawar, Pakistan; 3 Dr. James Degnan, PhD. Adjunct Assistant Professor, Senior Director (r) for Measurement and Institutional Research, Temple University, Philadelphia, USA

**Keywords:** Microteaching, Feedback, Postgraduate, Research Faculty

## Abstract

Multiple attributes are expected of postgraduate research supervisors. Provision of timely and effective face-to-face feedback is one such skill that carries enormous significance in supervisee’s professional development. Feedback allows the supervisees to improve upon their performances. Unfortunately, both supervisors and supervisees have contrasting approaches towards the ongoing feedback practices. This incongruence is attributed, in part, to a lack of structured pedagogic training among the medical professionals. A standardized schema is therefore required to acquire and harmonize this pedagogical skill. One such systemized way is a training method called microteaching. Microteaching has long been used to enhance and incorporate old and new undergraduate teaching skills, respectively. Here we propose a similar structured approach of micro-feedback to inculcate effective feedback skills among postgraduate research supervisors using feedback-based scenarios, simulated students, standardized checklists and audiovisual aids. Thus, micro-feedback exercise may prove to be quite promising in improving feedback practices of postgraduate research supervisors.

## INTRODUCTION

A range of distinctive characteristics is inherently desired of research supervisors to perform their roles effectively. Of all these skills, provision of a timely face-to-face feedback is considered as an essential constituent of effective supervision. In an academic context, feedback is not just a mean to inform the trainees about their performance rather it provided with an aim to improve it further.^1^ Several studies have defined feedback in a more holistic way by outlining various principles of good feedback practices.^2^,^3^ Feedback given on a supervisees’ performance not only assists in the research process and methodological skills but it also serves as a source of motivation and direction; thus allowing supervisees to improve upon their practices and take full responsibility for their progress. Feedback can be considered as a means to deepen a supervisees’ understanding of the problem under consideration and an opportunity for self-assessment. Furthermore, supervisees usually perceive feedback as a yardstick to measure the interim progress towards achieving their academic goals.

Unfortunately, there are conflicting views among the faculty and the students regarding feedback practices. Supervisors are of the view that for feedback to be effective, a supervisee needs to comprehend it effectively and be able to work upon it while supervisees are mostly not satisfied with the quality of feedback they receive.^2^ The disconnect between supervisors’ and supervisees’ perceptions can be attributed to a number of factors such as poor feedback practices and incongruity between supervisors and supervisees’ understanding of the purpose for feedback.^4^ Moreover, medical professionals, especially postgraduate research supervisors, are often not trained in pedagogic skills and therefore their capacity to teach and supervise is largely based mostly on trial and error, innate motivation, self-training or by observing others. At times, the complexity of teaching and supervisory situations can become quite overwhelming. Hence, standardized schemata for acquiring didactic skills are needed to harmonize the pedagogical skills. One such systemized way of assessing teaching skills is through a training method called microteaching.^5^ The term microtraining has been used in social and management sciences however, in educational research the micro skill sessions are usually employed to enhance teaching skills and hence the name microteaching is used.

### Using Microteaching for improving feedback skills

Microteaching is a teaching encounter carried out to develop new teaching skills and enhance old ones. Microteaching has been defined as “*an opportunity to develop and improve teaching skills with a small group of pupils (5 to 7) by means of a brief (5 minutes) single concept lesson”*.^6^ Thus the term “Micro” symbolizes not only a reduced class size and lesson duration but is also an indication of more precise observation during which special emphasis is given on an explicit teaching behavior.^7^ Microteaching has been utilized to train faculty on conventional as well as contemporary teaching skills.^8^ The microteaching sessions have long been used as a productive vehicle to determine the teaching behavior.

There is a systematic way of preparing and conducting the microteaching session.[5] It begins with defining the main objectives of the session. A microteaching session usually takes place in a classroom setting. The duration of the whole session depends on the total number of participants. The individual training session, lasting for 10 minutes for each participant, is carried out usually face-to-face but online or blended sessions can also be arranged. In these sessions, the participants are allotted different teaching scenarios. With the assistance of standardized trained students, the participant is asked to respond to the situation presented in the scenario. The activity is videotaped and recorded for deliberation. The remaining participants are provided with a micro-training checklist to rate their peer whose skill is being assessed. At the end of each training session, the recorded video is played again and the participant is asked to do self-evaluation. Furthermore, the colleagues are asked to deliberate on participants’ performance and provide effective feedback including constructive criticism and positive reinforcement.^7^,^8^

### Micro-Feedback Exercise

Keeping in mind the significance of effective feedback, the gap in the feedback practices and the utility of microteaching exercise, here we propose an amalgamation of both practices in the form of **“Micro-feedback exercise”**. As the name suggests, the microteaching method can be utilized to train and assess the face-to-face feedback practices of the faculty. This technique will include a focused emphasis only on the feedback practices of the faculty members. Standardized scenarios based on various feedback situations and corresponding checklists can be synthesized following any of the available effective feedback models or strategies such as Pendleton’s model or sandwich technique, in which a praise-critique-praise approach is used to allow a holistic coverage of major issues pertaining to feedback practices (Table-I).^9^

**Table-I:** Checklist for Micro-feedback session.

*Feedback Skills to be Judged during Microteaching session*	*Yes of Course*	*Somehow*	*Not Really*
Did the supervisor attract supervisee’s attention?	◯	◯	◯
Were the objectives of the feedback session achieved?	◯	◯	◯
Voice quality (loudness, tone, speed) was good?	◯	◯	◯
Body language was appropriate?	◯	◯	◯
The use of movement of the supervisor was purposeful?	◯	◯	◯
Did the supervisor ask questions? (Focused, pausing, cues, probing)	◯	◯	◯
Did the supervisor use positive reinforcement techniques?	◯	◯	◯
Did the feedback approach adopted by the supervisor fit the purpose?	◯	◯	◯
Did the supervisor interact adequately with the supervisee?	◯	◯	◯
Did the supervisor motivate the supervisee?	◯	◯	◯
Was the feedback session structured?	◯	◯	◯
Message of the supervisor was clear?	◯	◯	◯
Did the feedback session meet the supervisee’s needs?	◯	◯	◯
The feedback provided by the supervisor was constructive?	◯	◯	◯
The feedback provided by the supervisor was corrective?	◯	◯	◯
Was the feedback session descriptive rather than evaluative?	◯	◯	◯
Did the supervisor encourage the supervisee?	◯	◯	◯
Did the supervisor asked for supervisee’s reaction to feedback?	◯	◯	◯
Did the supervisor acknowledge the efforts of the supervisee?	◯	◯	◯
Did the session end with an action plan for future?	◯	◯	◯

There are multiple advantages of microteaching. It has widely been accepted as an effective teaching training tool by educators.^10^ Microteaching provides an opportunity for instructors to put themselves under the microscope in front of their peers and to be receptive towards the critique of how feedback in their teaching practices. In addition, microteaching allows the training to take place in an environment that may be is more comfortable than real life settings. It gives the learner an opportunity to get immediate feedback from senior and more experienced supervisors and work upon their appraisal to enhance their educational skills.^10^ Micro-feedback provides faculty members with an opportunity to review their practices, get an objective insight into it and adjust their practices accordingly in the light of peers’ opinions. This can be quite beneficial not just for the supervisors but also for their supervisees.^10^

## References

[1] Cleary ML, Walter G (2010). Giving feedback to learners in clinical and academic settings:Practical considerations. J Contin Educ Nurs.

[2] Handley K, Donovan BO, Price M, Handley K, Millar J, Donovan BO (2010). Feedback:All that effort, but what is the effect ?. Assess Eval High Educ.

[3] Juwah C, Macfarlane-Dick D, Matthew B, Nicol D, Ross D, Smith B (2004). Enhancing student learning through effective formative feedback [Internet]. The Higher Education Academy.

[4] Poertner S, Miller KM, Miller KM (1996). The art of giving and receiving feedback First.

[5] Hassan BA (1996). The Effects of Microteaching Supervisory Feedback on EFL Student Teacher Performance. Proceedings of the 15th National Symposium on English Language Teaching in Egypt Cairo:CDELT, Ain Shams University.

[6] Barth JL, Shermis SS (1984). Methods of instruction in social studies education.

[7] Douglass JE, Pfeiffer IL (1971). Microteaching as a practicum for supervisor education:The effect on supervisor conference behavior and skills.

[8] Vries PD (2008). Microtraining as a support mechanism for informal learning. e-learning Pap [Internet].

[9] Cantillon P, Sargeant J (2008). Giving feedback in clinical settings. Brit Med J[Internet].

[10] Meier JH (1968). Rationale for and Application of Microtraining To Improve Teaching. J Teach Educ [Internet].

